# Circular RNA_PDHX Promotes the Proliferation and Invasion of Prostate Cancer by Sponging MiR-378a-3p

**DOI:** 10.3389/fcell.2020.602707

**Published:** 2021-01-28

**Authors:** Yuanshen Mao, Wenfeng Li, Bao Hua, Xin Gu, Weixin Pan, Qi Chen, Bin Xu, Chao Lu, Zhong Wang

**Affiliations:** Department of Urology, Shanghai Ninth People’s Hospital, Shanghai Jiao Tong University School of Medicine, Shanghai, China

**Keywords:** prostate cancer, circPDHX, miR-378a-3p, IGF1R, growth, invasion

## Abstract

The dysregulation of circular RNAs (circRNAs) is implicated in the pathogenesis of prostate cancer (PCa). However, the underlying mechanisms by which hsa_circ_0003768 (circPDHX) contributes to PCa remain elusive. The differentially expressed circRNAs between PCa and normal tissues were identified by Gene Expression Omnibus dataset. The association of circPDHX and miR-378a-3p expression with the clinicopathological parameters and prognosis in patients with PCa was analyzed by fluorescence *in situ* hybridization and The Cancer Genome Atlas dataset. 3-(4,5-Dimethylthiazol-2-yl)-2,5-diphenyltetrazolium bromide (MTT) and Transwell assays as well as a xenograft tumor model were used to assess the role of circPDHX in PCa cells. circPDHX-specific binding with miR-378a-3p was validated by bioinformatic analysis, luciferase gene reporter, and RNA immunoprecipitation assays. As a result, we found that increased expression of circPDHX was associated with Gleason score (*P* = 0.001) and pathogenic T stage (*P* = 0.01) and acted as an independent prognostic factor of poor survival (*P* = 0.036) in patients with PCa. Knockdown of circPDHX inhibited cell proliferation and invasion *in vitro* and *in vivo*, but ectopic expression of circPDHX reversed these effects. Furthermore, circPDHX could sponge miR-378a-3p to promote cell proliferation, but miR-378a-3p counteracted circPDHX-induced cell proliferation and insulin-like growth factor 1 receptor (IGF1R) expression in PCa cells. In conclusion, our findings demonstrated that circPDHX facilitated the proliferation and invasion of PCa cells by sponging miR-378a-3p.

## Introduction

The morbidity of prostate cancer (PCa) ranks the first place, and it is a second cause of cancer-related mortality in male cancers in the United States (Siegel et al., 2017). Although more than 90% patients with PCa can be cured after surgical resection with a higher 5-year survival rate (Vis et al., 2006), the advanced cases still harbor an unfavorable prognosis due to the tumor dissemination and metastasis (Huang E. Y. et al., 2018). The aberrant expression of non-coding RNAs is associated with the pathogenesis of PCa (Shukla et al., 2016; Dong et al., 2018; Huang W. et al., 2018). Therefore, identification of novel biomarkers is urgently needed to increase the early detection of PCa.

Circular RNAa (circRNAs) as a new subgroup of non-coding RNAs (ncRNAs) have covalently closed loop structures and more tissue stability when compared with the corresponding linear RNAs due to their resistance to RNase R (Qu et al., 2017). Increasing data indicate that aberrant expression of circRNAs is involved in the progression of PCa. Low expression of circ-ITCH is associated with pathologic T stage, lymph node metastasis, and poor survival in patients with PCa (Huang E. et al., 2019). Upregulation of circSMARCA5, hsa_circ_102004, and hsa_circ_0004870 favors the proliferation and enzalutamide resistance in PCa cells (Kong et al., 2017; Greene et al., 2019; Si-Tu et al., 2019), but hsa_circ_0001206 inhibits the proliferation and invasion of PCa cells (Song et al., 2019). Moreover, circFOXO3 acts as an oncogene by sponging miR-29a-3p (Kong et al., 2020), while circUCK2 functions as a tumor suppressor in PCa by sponging miR-767-5p (Xiang et al., 2019). Until now, the functional role of hsa_circ_0003768 in PCa remains undocumented.

MicroRNAs (miRNAs) as another subtype of ncRNAs act a crucial role in the tumorigenesis of PCa (Chen L. et al., 2019; Huang S. et al., 2019; Zhao et al., 2019), of which miR-378a-3p is initiated to act as a tumor suppressor in rhabdomyosarcoma (Megiorni et al., 2014) and facilitates tamoxifen sensitivity in breast cancer by targeting GOLT1A (Ikeda et al., 2015). In addition, miR-378a-3p represses myoblasts growth in skeletal muscle development by targeting HDAC4 (Wei et al., 2016) and the activation of hepatic stellate cells by targeting Gli3 (Hyun et al., 2016). The serum levels of miR-378-3p are decreased in PCa (Nguyen et al., 2013) and may be used as a therapeutic strategy for PCa.

Herein, we identified a differentially expressed hsa_circ_0003768 (circPDHX) between PCa and normal tissue samples and found that elevated expression of circPDHX was associated with Gleason score, pathogenic T stage, and poor survival in patients with PCa; circPDHX contributed to the PCa tumorigenesis by sponging miR-378a-3p and might provide a potential biomarker for PCa.

## Materials and Methods

### Clinical Samples

The differentially expressed circRNAs between PCa and normal tissues were downloaded from the Gene Expression Omnibus (GEO) dataset^1^. The tissue microarray (No. XT16-016) including 75 paired PCa samples was purchased from Alenabio Biotechnology (Xi’an, China). The clinicopathological data of PCa patients as well as the expression levels of miR-98-5p, miR-99a-5p, miR-99b-5p, miR-100-5p, miR-182-5p, miR-378a-3p, miR-494-3p, let-7a-5p, let-7b-5p, let-7c-5p, let-7d-5p, let-7e-5p, let-7f-5p, let-7g-5p, let-7i-5p, and insulin-like growth factor 1 receptor (IGF1R) were downloaded from The Cancer Genome Atlas (TCGA) dataset^2^. The patients did not receive any chemotherapy, and the protocols were approved by the Ethics Committee of Shanghai Ninth People’s Hospital.

### Fluorescence *in situ* Hybridization

The probe sequence for circPDHX (5′-TGGCTGTGGCAACAGATAAA-3′) and biotin-labeled probe sequences for miR-378a-3p (5′-ACACAGGACCTGGAG TCAGGAG-3′) were used to analyze the expression of circPDHX and miR-378a-3p in PCa tissue samples. The detailed description of fluorescence *in situ* hybridization (FISH) analysis was conducted as previously reported (Dong et al., 2018).

### Plasmid Construction

The wild-type (WT) or mutant (Mut) 3′ untranslated region (UTR) vectors of circPDHX and IGF1R, containing miR-378a-3p binding sites, were constructed by annealing double-stranded DNA and inserting it into the pmirGLO vector at the *Bam*HI and *Eco*RI sites. Lentivirus mediated si-circPDHX (5′-GACTCTGTAAAGGTTGAAGAA-3′) or its negative control (si-NC) was constructed by Genechem (Shanghai, China), and circPDHX plasmids as well as miR-378a-3p mimic or inhibitor were offered by GenePharma (Shanghai, China).

### Cell Culture

PCa cell lines (PC3 and 22RV1) used in these studies were provided by the Institute of Chemistry and Cell Biology (Shanghai, China) and were cultured in Dulbecco’s modified Eagle’s medium (DMEM) medium supplemented with 10% heat-inactivated fetal bovine serum (FBS) in a humidified atmosphere containing 5% CO_2_ at 37°C.

### Quantitative Real-Time PCR

RNA was isolated from PCa cells using Trizol reagent (Invitrogen). One Step SYBR^®^ PrimeScript^TM^ PLUS RT-PCR Kit (TaKaRa, Beijing, China) was used to examine the expression of circPDHX and IGF1R. TaqMan^®^ MicroRNA Reverse Transcription Kit and TaqMan Universal Master Mix II (Thermo Fisher Scientific, Runcorn, United Kingdom) were used to measure miR-378a-3p expression. U6 or β-actin was used as an internal parameter. The 2^–ΔΔCT^ equation was used to quantify the data in triplicate. The primer sequences of circPDHX, miR-378a-3p, and IGF1R were indicated in Supplementary Table 1.

### Western Blot Analysis

Prostate cancer cell lines were harvested, and protein was extracted using RNA immunoprecipitation assay (RIPA) lysis buffer (Beyotime) and protease inhibitor (Beyotime). Primary antibodies against IGF1R (Ab-1161, Signalway, Shanghai, China) and glyceraldehyde 3-phosphate dehydrogenase (GAPDH) (ab9485, Abcam) were diluted (1:1,000) and incubated overnight at 4°C. Secondary antibody of goat anti-rabbit immunoglobulin G (IgG) (ab6721, Abcam, 1:10,000) was cultured for 1 h at room temperature. After rinsing, the polyvinylidene fluoride (PVDF) membrane of the antibodies was transferred onto the system. Captured signal was quantified by Image Lab Software 3.0 (Bio-Rad), and GAPDH was used as an internal parameter.

### MTT and Transwell Assays

Cell viability and invasive potential were assessed by 3-(4,5-dimethylthiazol-2-yl)-2,5-diphenyltetrazolium bromide (MTT) and Transwell assays according to the previous report (Dong et al., 2018).

### Actinomycin D and RNase R Treatment

Transcription was prevented by the addition of 2 mg/ml actinomycin D, and dimethyl sulfoxide (DMSO) (Sigma-Aldrich, St. Louis, MO, United States) was used as the control group. Total RNA was incubated for 30 min at 37°C with 3 U/μg of RNase R (Epicentre Technologies, Madison, WI, United States).

### Dual-Luciferase Reporter Assay

PCa cells were seeded into 24-well plates, and pmirGLO report vectors containing WT or Mut 3′UTR of circPDHX and IGF1R were cotransfected with miR-378a-3p mimic or inhibitor into PC3 and 22RV1 cell lines. After the transfection for 48 h, luciferase activities were detected with a dual-luciferase reporter system (Promega, Madison, WI, United States).

### RNA Immunoprecipitation

RNA immunoprecipitation (RIP) assay was conducted using a Magna RIP RNA-Binding Protein Immunoprecipitation Kit (Millipore, Billerica, MA, United States) according to the manufacturer’s instructions.

### Animal Experiments

Six-week-old female immune-deficient nude mice (BALB/c-nu) were injected subcutaneously with 5 × 10^7^ PC3 cells stably transfected with si-circPDHX or si-NC. Mice were monitored daily and developed a subcutaneous tumor. The tumor volume was detected every other day by using a formula: volume = length × width^2^/2. This study was approved by Animal Ethics Committee of our hospital.

### Statistical Analysis

Statistical analyses were conducted by SPSS 20.0 (IBM, SPSS, Chicago, IL, United States) and GraphPad Prism. Student’s *t*-test or chi-square test was used to assess the statistical significance for comparisons of two groups. Pearson correlation analysis was used to analyze the correlations. Overall survival curve was analyzed with the Kaplan–Meier method and log-rank test. Univariate and multivariate analyses were implemented by a Cox proportional hazard regression model. *P* < 0.05 was considered statistical significance.

## Results

### Identification of a Novel CircPDHX in PCa Cells

**FIGURE 1:**
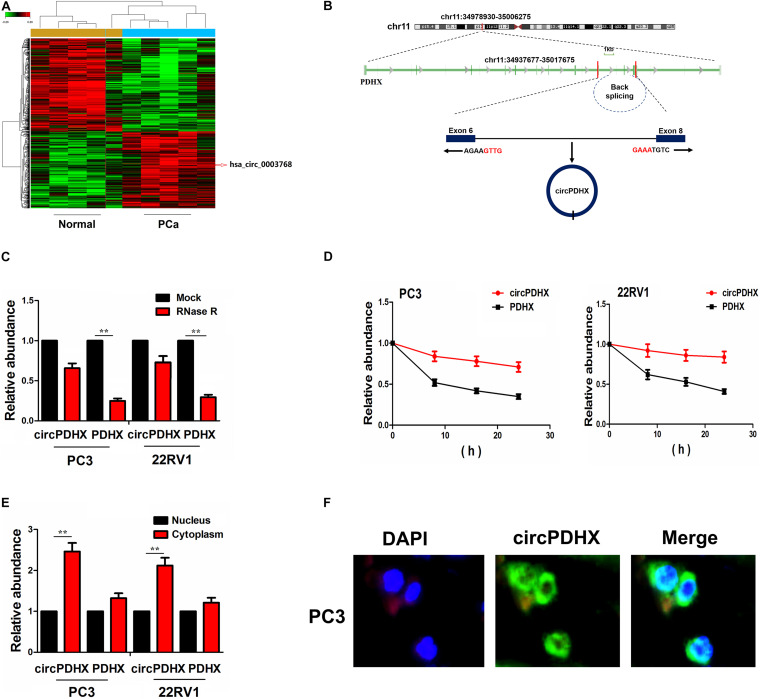
Identification of a novel circPDHX in prostate cancer (PCa) cells. **(A)** Gene Expression Omnibus (GEO) dataset analysis of the differentially expressed circular RNAs (circRNAs) between PCa and normal tissues. **(B)** The genomic loci of the PDHX gene and circPDHX. Arrows represent divergent primers that bind to the genomic region of circPDHX. **(C)** Quantitative real-time PCR (qRT-PCR) analysis of circPDHX and PDHX expression levels after treatment with RNase R in PC3 and 22RV1 cells. **(D)** qRT-PCR analysis of the half-life of circPDHX and PDHX after treatment with actinomycin D in PC3 and 22RV1 cells. **(E,F)** qRT-PCR and FISH analysis of the localization of circPDHX in PC3 and 22RV1 cells.

A microarray chip (GSE113124) was used to screen differentially expressed circRNAs between PCa and normal tissues. With the criteria of *P* < 0.01 and FC > 1.5, 546 downregulated circRNAs and 432 upregulated circRNAs were identified in PCa tissue samples, of which hsa_circ_0003768 had a significantly increased expression in PCa tissues (FC = 2.63, *P* = 0.0009; Figure 1A).

We found that hsa_circ_0003768 (chr11:34978930-35006275) originated from exon 6, eight regions within the pyruvate dehydrogenase complex component X (PDHX) locus and is termed as circPDHX (Figure 1B). Compared with linear PDHX, circPDHX gave rise to a resistance to RNase R treatment in PC3 and 22RV1 cell lines, and circPDHX had a loop structure in PCa cells (Figure 1C). After PC3 and 22RV1 cell lines were treated by a transcription inhibitor actinomycin D, quantitative real-time PCR (qRT-PCR) analysis showed that the half-life of circPDHX reached more than 24 h, but that of PDHX was <6 h in these two cells (Figure 1D). qRT-PCR and FISH analysis revealed that circPDHX was mainly localized in the cytoplasm of PCa cells (Figures 1E,F).

### Elevated Expression of CircPDHX Was Associated With Poor Survival in Patients With PCa

**FIGURE 2:**
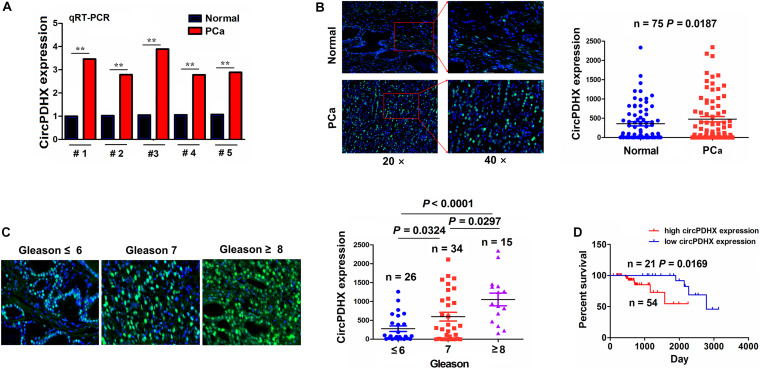
Increased expression of circPDHX was associated with poor survival in patients with prostate cancer (PCa). **(A)** Quantitative real-time PCR (qRT-PCR) analysis of the expression levels of circPDHX in PCa and normal tissues (*n* = 5). **(B)** Fluorescence *in situ* hybridization (FISH) analysis of the expression levels of circPDHX in pair-matched PCa tissue samples (*n* = 75). **(C)** FISH analysis of the expression levels of circPDHX in PCa tissues with different Gleason scores. **(D)** Kaplan–Meier analysis of the association of high or low circPDHX expression with overall survival in patients with PCa.

The expression of circPDHX was examined in PCa tissues by qRT-PCR analysis, which indicated that circPDHX expression levels were increased in PCa tissue samples as compared with the pair-matched normal tissues (*P* < 0.01; Figure 2A). This result was further validated by FISH analysis in paired 75 PCa samples (*P* = 0.0187; Figure 2B). We then analyzed the association of circPDHX expression with Gleason scores in PCa and found that circPDHX expression levels were elevated gradually with increased Gleason scores (*P* < 0.05; Figure 2C).

According to circPDHX expression levels, survival time, and status, cutoff value (495.56), area under curve (AUC) (0.64), sensitivity (80.0%), and specificity (58.7%) of circPDHX were achieved in patients with PCa (Supplementary Figure 1) by using a cutoff finder^3^. As indicated in Supplementary Table 2, the elevated expression of circPDHX was positively associated with Gleason score (*P* = 0.001) and pathogenic T stage (*P* = 0.01) in patients with PCa. Kaplan–Meier analysis demonstrated that the patients with high circPDHX expression harbored a poorer survival as compared with those with low circPDHX expression (*P* = 0.0169; Figure 2D). Univariate and multivariate analyses unveiled that high circPDHX expression as well as higher Gleason score was an independent prognostic factor of poor survival in PCa patients (*P* = 0.036; Supplementary Table 3).

### CircPDHX Promoted Proliferation, Colony Formation, and Invasion of PCa Cells

**FIGURE 3:**
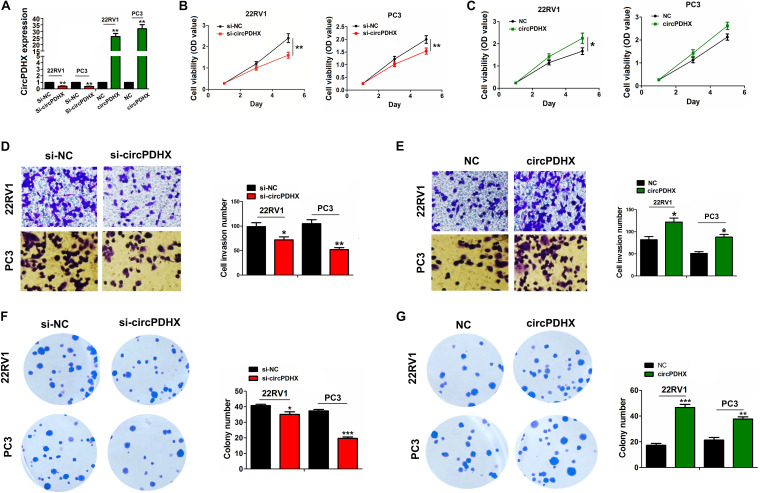
circPDHX promoted prostate cancer (PCa) cell proliferation, colony formation, and cell invasion. **(A)** Quantitative real-time PCR (qRT-PCR) analysis of the expression levels of circPDHX after transfection with si-circPDHX or circPDHX plasmids in 22RV1 and PC3 cell lines. **(B,C)** 3-(4,5-Dimethylthiazol-2-yl)- 2,5-diphenyltetrazolium bromide (MTT) analysis of the cell proliferation after transfection with si-circPDHX or circPDHX plasmids in 22RV1 and PC3 cell lines. **(D,E)** Transwell analysis of cell invasion after transfection with si-circPDHX or circPDHX plasmids in 22RV1 and PC3 cells. **(F,G)** Colony formation analysis of the cell colony formation abilities after transfection with si-circPDHX or circPDHX plasmids in 22RV1 and PC3 cell lines. Data shown are the mean ± SEM of three experiments. *P < 0.05; **P < 0.01.

Elevated expression of circPDHX in PCa tissues indicated it as a tumor-promoting factor. To confirm this hypothesis, we assessed the functional role of circPDHX in PCa cells. The silencing efficiency of si-circPDHX or overexpression efficiency of circPDHX plasmids was confirmed in 22RV1 and PC3 cells by qRT-PCR analysis (Figure 3A). Consequently, we found that knockdown of circPDHX repressed cell viability (Figure 3B), invasive potential (Figure 3D), and colony formation (Figure 3F) in 22RV1 and PC3 cells, but restored circPDHX expression reversed these effects (Figures 3C,E,G).

### CircPDHX Was Negatively Associated With MiR-378a-3p Expression in PCa Tissues

**FIGURE 4:**
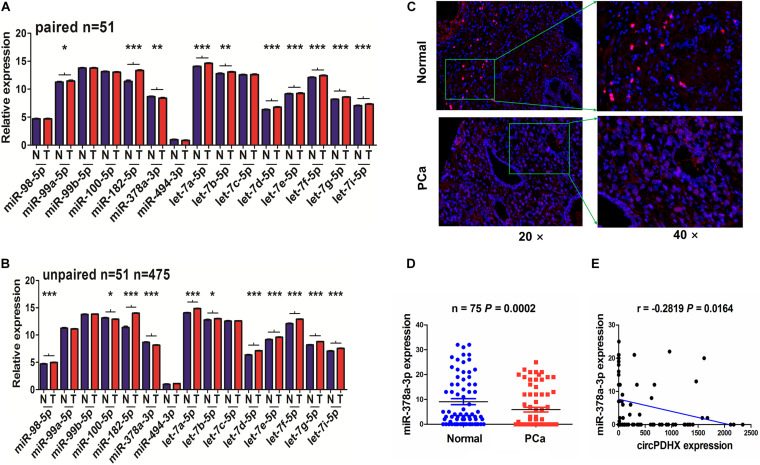
circPDHX had a negative correlation with miR-378a-3p expression in prostate cancer (PCa) tissue samples. **(A,B)** The Cancer Genome Atlas (TCGA) analysis of the expression levels of 15 miRNAs in paired (*n* = 51) and unpaired PCa tissue samples (*n* = 475). **(C,D)** Fluorescence *in situ* hybridization (FISH) analysis of the expression levels of miR-378a-3p in pair-matched PCa tissue samples (*n* = 75). **(E)** Pearson correlation analysis of the correlation of circPDHX with miR-378a-3p expression in PCa tissues.

To elucidate the underlying mechanisms of circPDHX in PCa cells, we identified 15 miRNAs that may have the potential to bind with circPDHX by using Circular RNA Interactome^4^ and found that only miR-378a-3p had a decreased expression in paired (*n* = 51, Figure 4A) and unpaired PCa samples (*n* = 475, Figure 4B). Reduced expression of miR-378a-3p was further validated by FISH analysis in PCa samples (*n* = 75, *P* = 0.0002; Figures 4C,D). Pearson correlation analysis indicated that miR-378a-3p possessed a negative correlation with circPDHX expression in PCa samples (*r* = -0.2819, *P* = 0.0164; Figure 4E).

We then found that low expression of miR-378a-3p was associated with higher Gleason score (*P* = 0.041) and pathological N stage (*P* = 0.036) in PCa patients (Supplementary Table 4). However, the patients with low miR-378a-3p expression had no difference in poor survival and tumor recurrence as compared with those with high miR-378a-3p expression (Supplementary Figure 2).

### CircPDHX Could Sponge MiR-378a-3p in PCa Cells

**FIGURE 5:**
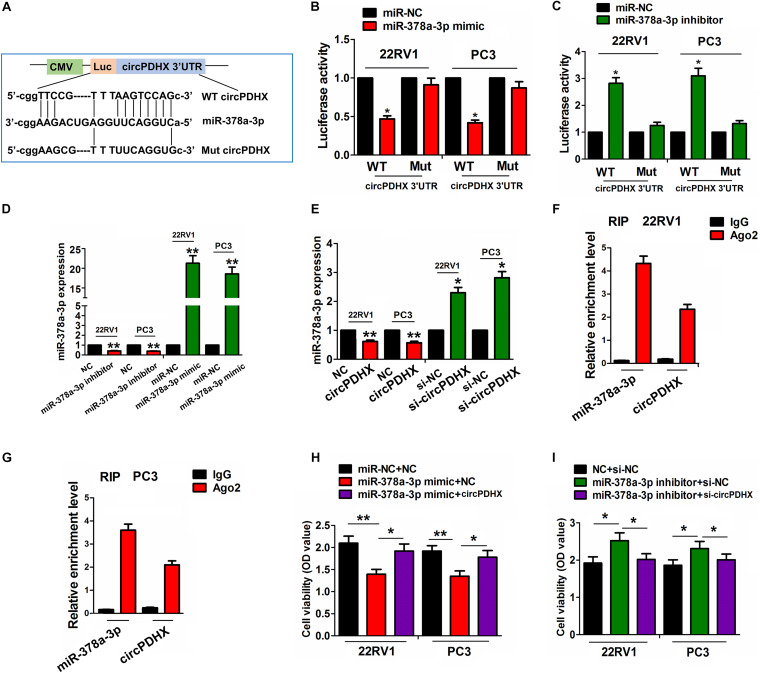
circPDHX negatively regulated miR-378a-3p expression in prostate cancer (PCa) cells. **(A)** Schematic representation of the binding sites of miR-378a-3p with WT or Mut circPDHX 3′ untranslated region (UTR). **(B,C)** Assessment of luciferase activities of WT or Mut circPDHX 3′UTR after cotransfection with miR-378a-3p mimic or inhibitor and WT or Mut circPDHX 3′UTR reporter vectors in 22RV1 and PC3 cells. **(D)** Quantitative real-time PCR (qRT-PCR) analysis of the expression levels of miR-378a-3p after transfection with miR-378a-3p inhibitor or mimic in 22RV1 and PC3 cell lines. **(E)** qRT-PCR analysis of the expression levels of miR-378a-3p after transfection with circPDHX plasmids or si-circPDHX in 22RV1 and PC3 cell lines. **(F,G)** RNA immunoprecipitation (RIP) analysis of the enrichment levels of circPDHX and miR-378a-3p pulled down from Ago2 protein in 22RV1 and PC3 cells. **(H,I)** 3-(4,5-Dimethylthiazol-2-yl)-2,5-diphenyltetrazolium bromide (MTT) analysis of cell proliferation after cotransfection with circPDHX plasmids and miR-378a-3p mimic or si-circPDHX and miR-378a-3p inhibitor in 22RV1 and PC3 cells. Data shown are the mean ± SEM of three experiments. *P < 0.05, **P < 0.01.

The binding sites of miR-378a-3p with WT or Mut circPDHX 3′UTR are indicated in Figure 5A. To confirm whether circPDHX could bind with miR-378a-3p, we cotransfected 22RV1 and PC3 cells with WT or Mut circPDHX 3′UTR reporter vectors and the miR-378a-3p mimic or inhibitor and found that miR-378a-3p mimic reduced the luciferase activities of WT circPDHX 3′UTR in 22RV1 and PC3 cell lines (Figure 5B), while the miR-378a-3p inhibitor increased their luciferase activities (Figure 5C). However, the miR-378a-3p mimic or inhibitor exerted no effects on those of Mut circPDHX 3′UTR as compared with the control group (Figures 5B,C). Further investigations showed that the knockdown or overexpression efficiency of the miR-378a-3p inhibitor or mimic in 22RV1 and PC3 cell lines was determined by qRT-PCR analysis (Figure 5D), but the miR-378a-3p mimic or inhibitor had no effect on circPDHX expression levels (Supplementary Figure 3). It was noted that the restored expression of circPDHX decreased the expression of miR-378a-3p, while silencing circPDHX increased its expression levels in these two cell lines (Figure 5E). Furthermore, RIP assay was conducted for Ago2 protein in 22RV1 and PC3 cells, and the expression of endogenous circPDHX and miR-378a-3p pulled down from Ago2-expressed 22RV1 and PC3 cells, indicated by qRT-PCR analysis, was enriched in Ago2 pellet in comparison with the input control (Figures 5F,G). After cotransfection with circPDHX plasmids and miR-378a-3p mimic or si-circPDHX and miR-378a-3p inhibitor in 22RV1 and PC3 cells, we found that the miR-378a-3p mimic inhibited cell viability and reversed circPDHX-induced cell proliferation (Figure 5H), while the miR-378a-3p inhibitor showed the opposite effects (Figure 5I).

### MiR-378a-3p Reversed CircPDHX-Induced IGF1R Expression in PCa Cells

**FIGURE 6:**
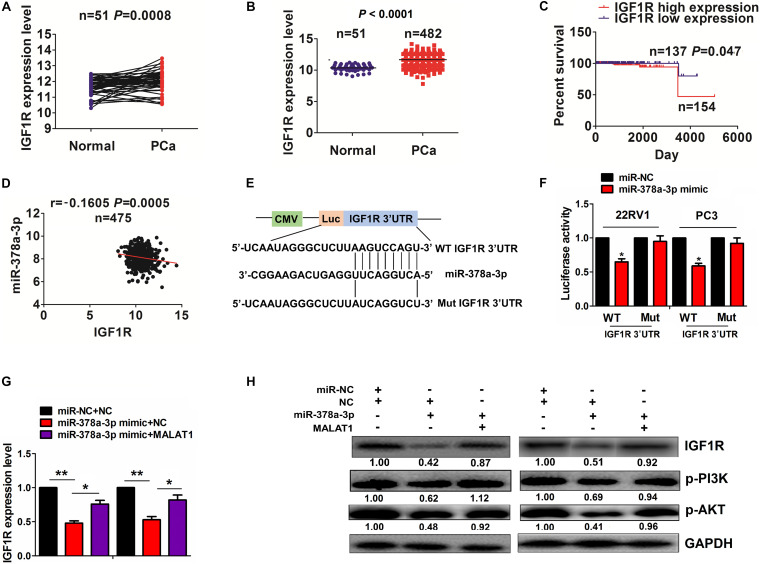
miR-378a-3p reversed circPDHX-induced insulin-like growth factor 1 receptor (IGF1R) expression in prostate cancer (PCa) cells. **(A,B)** The Cancer Genome Atlas (TCGA) analysis of the expression levels of IGF1R in paired (*n* = 51) and unpaired PCa tissue samples (*n* = 482). **(C)** Kaplan–Meier analysis of the association of high or low IGF1R expression with poor survival in patients with PCa. **(D)** Pearson correlation analysis of the correlation of IGF1R with miR-378a-3p expression in PCa tissues. **(E)** Schematic representation of the binding sites of miR-378a-3p and WT or Mut 3′ untranslated region (UTR) of IGF1R. **(F)** Estimation of the luciferase activities of WT or Mut IGF1R 3′ UTR after cotransfection with miR-378a-3p mimic and WT or Mut IGF1R 3′ UTR reporter vectors in 22RV1 and PC3 cells. **(G,H)** Quantitative real-time PCR (qRT-PCR) and Western blot analysis of IGF1R expression levels after cotransfection with circPDHX and miR-378a-3p mimic in 22RV1 and PC3 cells. Data shown are the mean ± SEM of three experiments. *P < 0.05; **P < 0.01.

We identified the targets of miR-378a-3p and found that miR-378a-3p might have the greatest potential to bind with 3′UTR of IGF1R by using the starBase v2.0 prediction tool (^5^, Supplementary Table 5). TCGA cohort showed that IGF1R expression was increased in paired (*n* = 51, *P* = 0.0008; Figure 6A) and unpaired PCa samples (*n* = 482, *P* < 0.0001; Figure 6B). Then, high expression of IGF1R was associated with pathological T stage in PCa patients (*P* = 0.034, Supplementary Table 6), and the patients with high IGF1R expression harbored a poorer survival (*P* = 0.047, Figure 6C) as compared with those with low IGF1R expression. However, univariate and multivariate analyses uncovered that high IGF1R expression was not an independent prognostic factor of poor survival in PCa patients (Supplementary Table 7).

Pearson correlation analysis indicated that miR-378a-3p had a negative correlation with IGF1R expression in PCa tissues (*r* = -0.1605, *P* = 0.0005; Figure 6D). The binding sites of miR-378a-3p with WT or Mut 3′ UTR of IGF1R were indicated in Figure 6E. To confirm whether miR-378a-3p could bind with 3′ UTR of IGF1R, we cotransfected 22RV1 and PC3 cell lines with WT or Mut IGF1R 3′ UTR reporter vectors and miR-378a-3p mimic and found that miR-378a-3p mimic reduced the luciferase activities of WT IGF1R 3′ UTR in these two cell lines but had no effects on those of Mut IGF1R 3′ UTR as compared with the miR-NC group (Figure 6F). qRT-PCR and Western blot analysis indicated that miR-378a-3p inhibited the activation of IGF1R/PI3K/AKT signaling and reversed cricPDHX-induced signaling activation in 22RV1 and PC3 cell lines (Figures 6G,H).

### Knockdown of CircPDHX Inhibited the Tumorigenesis of PCa *in vivo*

**FIGURE 7:**
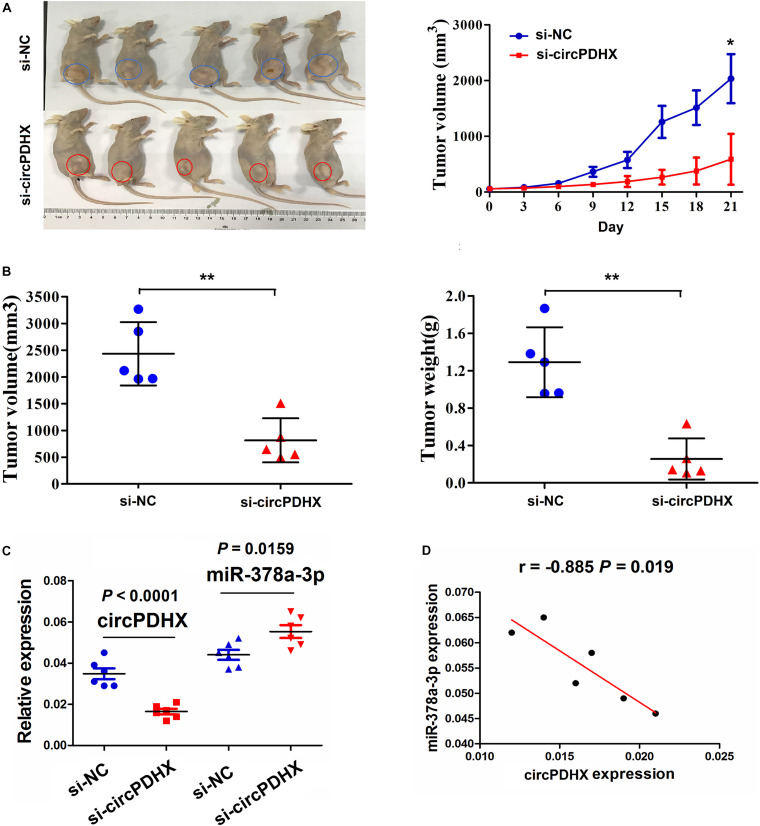
Knockdown of circPDHX suppressed PC3 xenograft tumor growth. **(A)** Representative photographs of the xenograft tumors and assessment of the tumor proliferation trend after treatment with si-circPDHX or si-NC transfected PC3 cells. **(B)** Comparison of PC3 xenograft tumor volume and weight between si-circPDHX and si-NC groups. **(C)** Quantitative real-time PCR (qRT-PCR) analysis of circPDHX and miR-378a-3p expression in si-circPDHX or si-NC treated PC3 tumor tissues. **(D)** Pearson correlation analysis of the correlation of circPDHX with miR-378a-3p expression in si-circPDHX-treated PC3 tumor tissues. Data shown are the mean ± SEM of three experiments. *P < 0.05; **P < 0.01.

To confirm the effects of circPDHX on PCa tumor growth *in vivo*, a xenograft tumor model was constructed after subcutaneous inoculation with si-circPDHX stably transfected PC3 cells. During the growth period, the length and weight of PCa tumors were measured. We found that the proliferative capabilities of PCa tumors were impeded by downregulation of circPDHX in comparison with si-NC group (Figure 7A). After PCa tumor tissues were harvested, the average tumor volume and weight were smaller in the si-circPDHX group than those in the si-NC group (Figure 7B). qRT-PCR analysis indicated that circPDHX expression was markedly reduced, while miR-378a-3p expression was increased in the si-circPDHX group as compared with the si-NC group (Figure 7C). Pearson correlation analysis showed that circPDHX had a negative correlation with miR-378a-3p expression in si-circPDHX group (Figure 7D).

## Discussion

circRNAs as a novel ncRNA have been implicated in the prognosis and progression of multiple malignancies including PCa (Wang et al., 2018; Zhang et al., 2019). The upregulation of circ-ITCH is associated with distant metastasis and poor prognosis in patients with PCa (Huang E. et al., 2019). Herein, we identified a differentially expressed circPDHX and found that high expression of circPDHX was associated with Gleason score and pathogenic T stage and acted as an independent prognostic factor of poor survival in PCa patients.

Previous studies showed that circRNAs can act as oncogenic factors (Kong et al., 2017; Si-Tu et al., 2019) or tumor suppressors in PCa (Song et al., 2019). Herein, we assess the functional role of circPDHX in PCa cells and found that silencing circPDHX repressed the cell viability, colony formation, and cell invasion *in vitro* and *in vivo*, but ectopic expression of circPDHX indicated the tumor-promoting effects. Our results indicated that circPDHX might act as an oncogene in PCa.

Increasing investigations demonstrated that circRNAs can act as miRNA sponges to participate in cancer regulation (Chen Y. et al., 2019). circHIPK3 and circZNF609 enhance the proliferation and invasion of PCa by sponging miR-193a-3p/-338-3p or miR-186-5p (Cai et al., 2019; Chen D. et al., 2019; Jin et al., 2019), whereas circRNA17 and circUCK2 suppress the progression of PCa by sponging miR-181c-5p or miR-767-5p (Wu et al., 2019; Xiang et al., 2019). Herein, we found that circPDHX could bind with Ago2-miR-378a-3p complex and negatively regulate miR-378a-3p expression in PCa cells. Likewise, hsa_circ_0007059 suppresses cell proliferation and epithelial–mesenchymal transition in lung cancer by sponging miR-378a-3p (Gao et al., 2019). These studies indicated that circPDHX might act as a sponge of miR-378a-3p to promote PCa progression.

**FIGURE 8:**
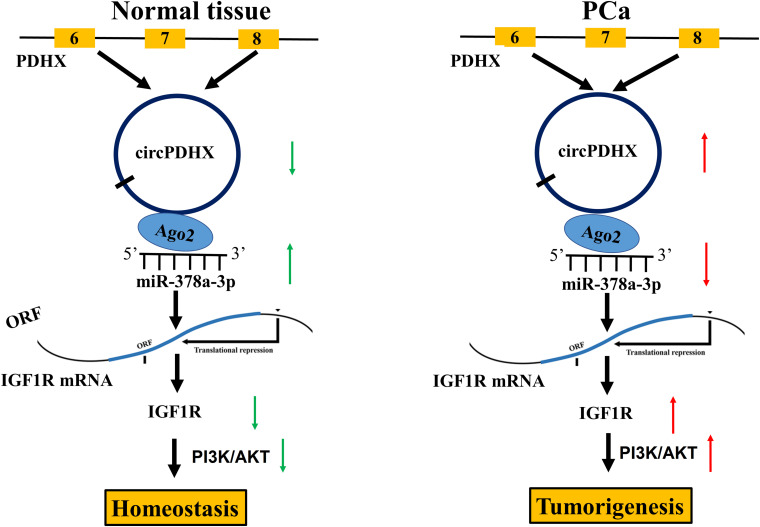
Schematic representation of the proposed mechanisms of circPDHX in prostate cancer (PCa). circPDHX acted as a sponge of miR-378a-3p to upregulate IGF1R expression, contributing to the tumorigenesis of PCa.

Some studies have shown that miR-378a-3p is downregulated in colorectal cancer (CRC), and its low expression indicates poor survival in patients with CRC (Li et al., 2014). It also restrains melanoma growth via targeting PARVA (Velazquez-Torres et al., 2018) and acts as a chemosensitizer in ovarian cancer (Xu et al., 2018). In accordance, we found that low expression of miR-378a-3p was associated with Gleason score and pathogenic N stage in patients with PCa. IGF1R was further validated as a direct target of miR-378a-3p in PCa cells and indicated a poor survival in patients with PCa. In addition, miR-378a-3p displayed a negative correlation with circPDHX expression and attenuated circPDHX-induced cell proliferation and IGF1R expression in PCa cells. Our results suggested that circPDHX might act as a sponge of miR-378a-35p to upregulate IGF1R expression, contributing to the tumorigenesis of PCa (Figure 8).

Taken together, the increased expression of circPDHX was associated with Gleason score and pathogenic T stage and acted as an independent prognostic factor of poor survival in patients with PCa. circPDHX facilitated the tumorigenesis of PCa by sponging miR-378a-3p. This study might offer a potential biomarker for the detection of PCa.

## Data Availability Statement

The original contributions presented in the study are included in the article/Supplementary Material, further inquiries can be directed to the corresponding author/s.

## Ethics Statement

The studies involving human participants were reviewed and approved by the protocols were approved by the Ethics Committee of Shanghai Ninth People’s Hospital. The patients/participants provided their written informed consent to participate in this study. The animal study was reviewed and approved by this study was approved by the Animal Ethics Committee of Shanghai Ninth People’s Hospital.

## Author Contributions

BX, CL, and ZW designed this study. YM and WL performed the experiments and wrote the manuscript. BX searched the literature. WP and QC collected the clinical samples. BH and XG assisted in conducting the experiments. CL revised the manuscript, and all authors read and approved the final manuscript. All authors contributed to the article and approved the submitted version.

## Conflict of Interest

The authors declare that the research was conducted in the absence of any commercial or financial relationships that could be construed as a potential conflict of interest.

## Footnotes

1 https://www.gcbi.com.cn/gclib/html/index

2 http://xena.ucsc.edu/

3 http://molpath.charite.de/cutoff/load.jsp

4 https://circinteractome.nia.nih.gov/index.html

5 http://starbase.sysu.edu.cn/starbase2/index.php
