# Supplementary figures and images for: Circular RNA_PDHX Promotes the Proliferation and Invasion of Prostate Cancer by Sponging MiR-378a-3p
Source: Front Cell Dev Biol. 2021 Jan 28;8:602707. doi: 10.3389/fcell.2020.602707 (PMC7901981; doi:10.3389/fcell.2020.602707)

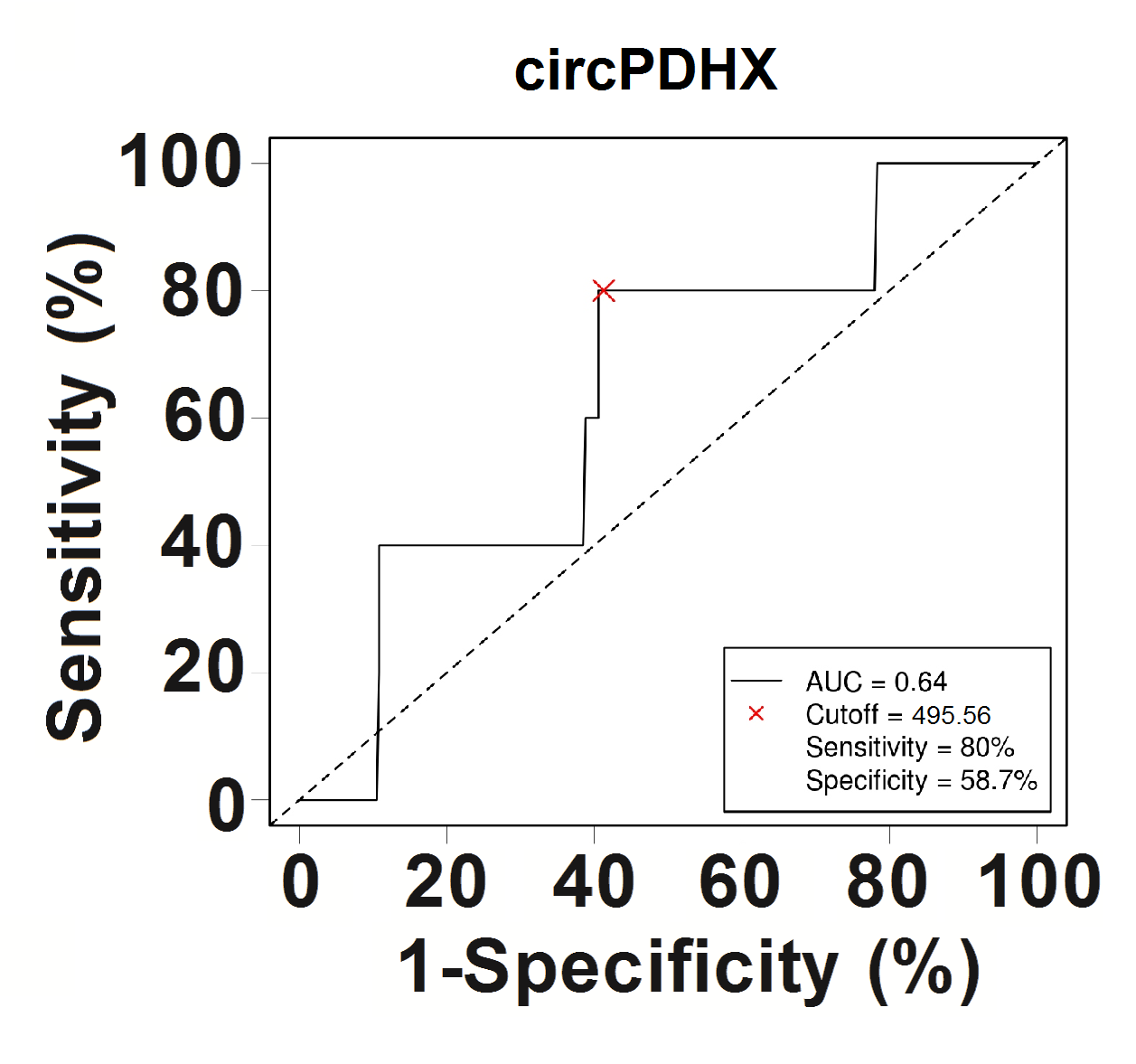

Supplement: Supplementary file 1 [file Image_1.TIF]

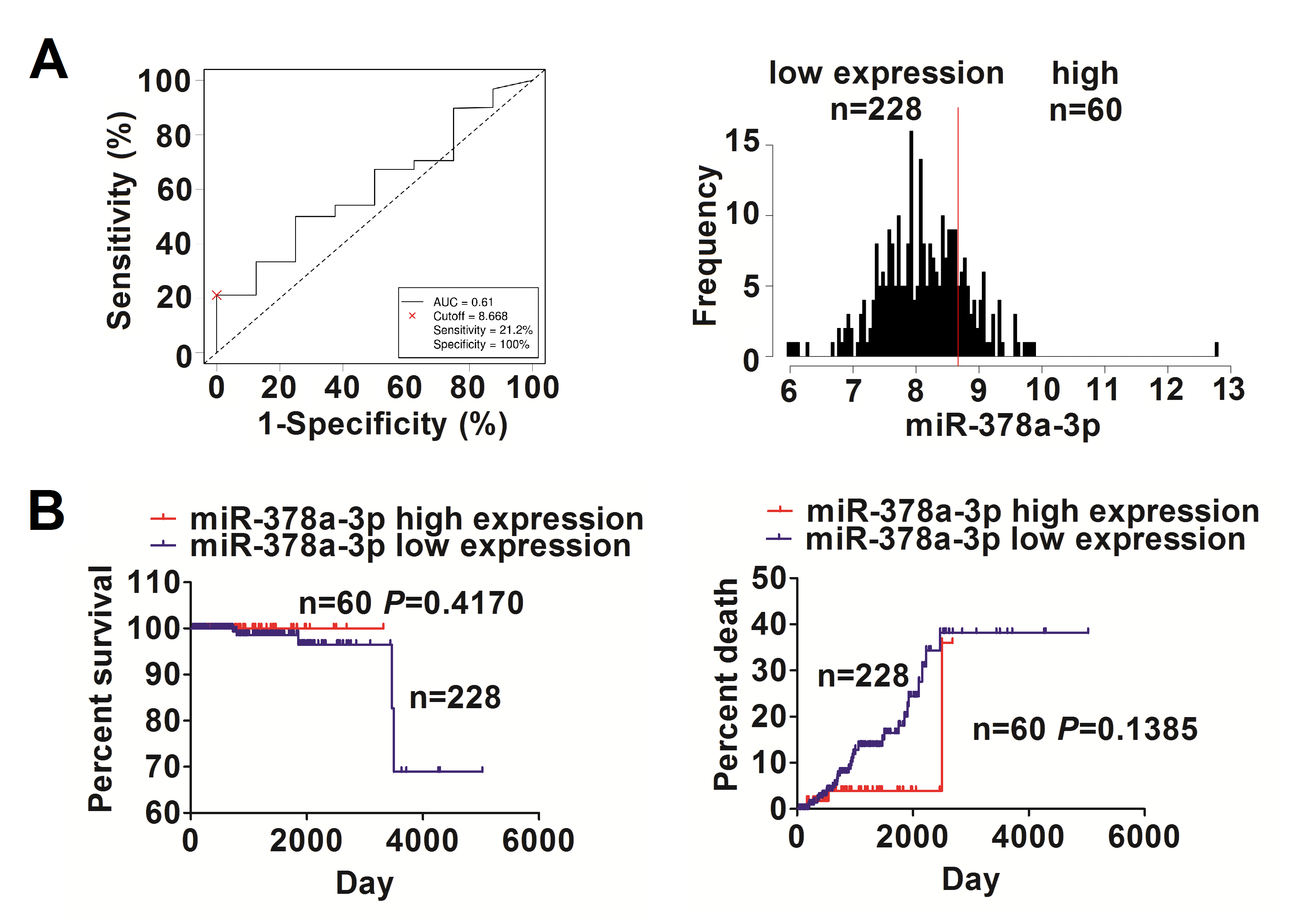

Supplement: Supplementary file 2 [file Image_2.TIF]

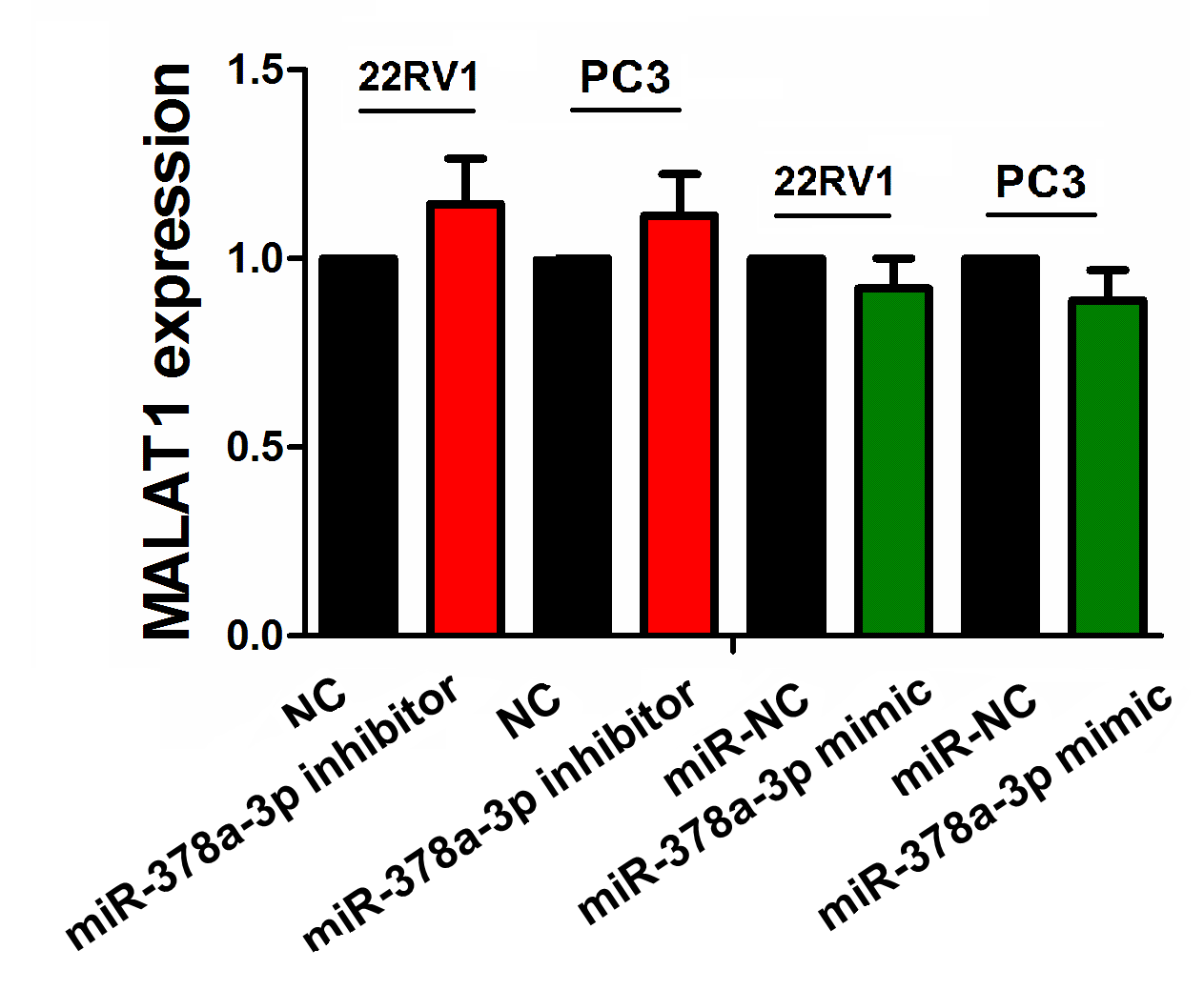

Supplement: Supplementary file 3 [file Image_3.TIF]
