# Supplementary material for: Circular RNA_PDHX Promotes the Proliferation and Invasion of Prostate Cancer by Sponging MiR-378a-3p
Source: Front Cell Dev Biol. 2021 Jan 28;8:602707. doi: 10.3389/fcell.2020.602707 (PMC7901981; doi:10.3389/fcell.2020.602707)
Supplement: Supplementary file 4 [file Table_1.DOCX]

**Supplementary Figure legends**

**Supplementary Figure S1** The cutoff value, AUC, specificity, and sensitivity of circPDHX were obtained according to circPDHX expression levels, overall survival time and survival status in PCa samples.

**Supplementary Figure S2** TCGA analysis of the association of miR-378a-3p expression with the prognosis in patients with PCa. (A) The cutoff value, AUC, specificity, and sensitivity of miR-378a-3p were obtained according to miR-378a-3p expression levels, overall survival time and survival status in PCa samples, and the patients were divided into high miR-378a-3p expression (*n* = 60) and low MALAT1 expression groups (*n* = 228) according to its cutoff value. (B) Kaplan-Meier analysis of the association of high or low miR-378a-3p expression with poor survival and tumor recurrence in patients with PCa.

**Supplementary Figure S3** qRT-PCR analysis of the expression levels of circPDHX after transfection with miR-378a-3p inhibitor or mimic in 22RV1 and PC3 cell lines.

**Supplementary Tables**

**Table S1.** The list of primer sequences

| Markers | Sense (5’-3’) | Antisense (5’-3’) |
| --- | --- | --- |
| circPDHX | TGGGAATTTCAACATGTTTCC | TGACATTTCAGTGGCTGTGG |
| β-actin | GCGTGACATTAAGGAGAAGC | CCACGTCACACTTCATGATGG |
| miR-378a-3p | ACUGGACUUGGAGUCAGAAGG |  |
| U6 | CAGCACATATACTAAAATTGGAACG | ACGAATTTGCGTGTCATCC |
| GAPDH | GGTGAAGGTCGGAGTCAACG | CAAAGTTGTCATGGATGHACC |
| IGF1R | ATGCTGACCTCTGTTACCTCT | GGCTTATTCCCCACAATGTAGTT |

**Table S2.** The association of circPDHX expression with clinicopathological

characteristics of PCa patients

| Variables | Cases  (n) | circPDHX | | *P* value |
| --- | --- | --- | --- | --- |
|  |  | High | Low |  |
| Total | 75 | 54 | 21 |  |
| *Age (years)* |  |  |  |  |
| ≥60 | 45 | 33 | 12 |  |
| <60 | 30 | 21 | 9 | 0.754 |
| *Gleason score* |  |  |  |  |
| 5-7 | 42 | 24 | 18 |  |
| 8-10 | 33 | 30 | 3 | 0.001 |
| *Pathologic T stage* |  |  |  |  |
| T1/T2 | 29 | 16 | 13 |  |
| T3/T4 | 46 | 38 | 8 | 0.01 |
| *Pathologic N stage* |  |  |  |  |
| Negative | 62 | 43 | 19 |  |
| Positive | 13 | 11 | 2 | 0.268 |
| *Pathologic M stage* |  |  |  |  |
| Negative | 72 | 52 | 20 |  |
| Positive | 3 | 2 | 1 | 0.835 |

**Table S3.** Cox regression analysis of circPDHX expression as survival predictor

| Variables | Univariate Cox regression analysis | |  | Multivariate Cox regression analysis | |
| --- | --- | --- | --- | --- | --- |
|  | RR (95% CI) | *P* value |  | RR (95% CI) | *P* value |
| *Age (years)* |  |  |  |  |  |
| <60 vs. ≥60 | 0.931 (0.473 to 1.834) | 0.836 |  | NA | NA |
| *Gleason score* |  |  |  |  |  |
| 5-7 vs. 8-10 | 8.219 (3.382 to 19.974) | <0.0001 |  | 5.905 (2.221 to 15.700) | <0.0001 |
| *Pathologic T stage* |  |  |  |  |  |
| T3+T4 vs. T1+T2 | 2.194 (1.614 to 4.897) | 0.035 |  | 1.719 (0.595 to 4.965) | 0.317 |
| *Pathologic N staging* |  |  |  |  |  |
| Positive vs. Negative | 2.294 (1.082 to 4.862) | 0.030 |  | 0.812 (0.364 to 1.814) | 0.612 |
| *Pathologic M stage* |  |  |  |  |  |
| Positive vs. Negative | NA | NA |  | NA | NA |
| *MALAT1 expression* |  |  |  |  |  |
| High VS. Low | 4.077 (1.851 to 9.108) | 0.021 |  | 2.172 (1.224 to 7.994) | 0.036 |

NA: not analyzed

**Table S4** The correlation of miR-378a-3p expression with clinicopathologic

characteristics of PCa patients

| Variables | Cases  (n) | miR-378a-5p | | *P* value |
| --- | --- | --- | --- | --- |
|  |  | High | Low |  |
| Total | 288 | 60 | 228 |  |
| *Age (years)* |  |  |  |  |
| ≥60 | 173 | 33 | 140 |  |
| <60 | 115 | 27 | 88 | 0.378 |
| *Gleason score* |  |  |  |  |
| 5-7 | 162 | 41 | 121 |  |
| 8-10 | 126 | 19 | 107 | 0.041 |
| *Clinical T stage* |  |  |  |  |
| T1/T2 | 244 | 54 | 190 |  |
| T3/T4 | 44 | 6 | 38 | 0.232 |
| *Pathologic T stage* |  |  |  |  |
| T1/T2 | 111 | 26 | 85 |  |
| T3/T4 | 177 | 34 | 143 | 0.456 |
| *Pathologic N stage* |  |  |  |  |
| Negative | 237 | 55 | 182 |  |
| Positive | 51 | 5 | 46 | 0.036 |
| *Pathologic M stage* |  |  |  |  |
| Negative | 287 | 60 | 227 |  |
| Positive | 1 | 0 | 1 | 1.000 |

**Table S5** Identification of the targets of miR-378a-3p

| geneName | position | targetScan | picTar | RNA22 | PITA | miRanda |
| --- | --- | --- | --- | --- | --- | --- |
| IGF1R | chr15:99506261-99506267[+] | 5202[7] | 5202[7] | 0[0] | 5202[7] | 0[0] |
| PRICKLE1 | chr12:42852962-42852983[-] | 0[0] | 0[0] | 0[0] | 0[0] | 61[5] |
| SURF6 | chr9:136197935-136197957[-] | 0[0] | 0[0] | 0[0] | 0[0] | 52[5] |
| DAZAP2 | chr12:51637283-51637288[+] | 0[0] | 602[9] | 0[0] | 602[9] | 1223[10] |
| DAZAP2 | chr12:51637450-51637455[+] | 0[0] | 1496[6] | 0[0] | 0[0] | 0[0] |
| RAB2B | chr14:21927904-21927924[-] | 0[0] | 0[0] | 0[0] | 0[0] | 162[5] |
| NUSAP1 | chr15:41672583-41672604[+] | 0[0] | 0[0] | 318[12] | 0[0] | 0[0] |
| RRP1B | chr21:45115587-45115594[+] | 157[5] | 0[0] | 0[0] | 0[0] | 162[6] |
| SOBP | chr6:107980580-107980600[+] | 0[0] | 0[0] | 0[0] | 0[0] | 111[7] |
| ADAR | chr1:154554630-154554649[-] | 0[0] | 0[0] | 0[0] | 0[0] | 73[6] |
| KIAA1267 | chr17:44108605-44108626[-] | 0[0] | 0[0] | 91[7] | 0[0] | 0[0] |
| RDX | chr11:110100972-110100993[-] | 0[0] | 0[0] | 525[6] | 0[0] | 0[0] |
| GOLIM4 | chr3:167727714-167727736[-] | 0[0] | 0[0] | 0[0] | 0[0] | 883[7] |
| PCGF3 | chr4:762540-762561[+] | 0[0] | 0[0] | 83[5] | 0[0] | 0[0] |
| FOXK2 | chr17:80559963-80559984[+] | 0[0] | 0[0] | 899[5] | 0[0] | 0[0] |
| SSFA2 | chr2:182794963-182794983[+] | 0[0] | 0[0] | 0[0] | 0[0] | 4940[10] |
| ZNF512B | chr20:62590008-62590029[-] | 0[0] | 0[0] | 1[5] | 0[0] | 0[0] |
| GPR180 | chr13:95279809-95279827[+] | 0[0] | 0[0] | 0[0] | 0[0] | 188[6] |
| OAZ1 | chr19:2271831-2271852[+] | 0[0] | 0[0] | 0[0] | 0[0] | 296[8] |
| RAB5B | chr12:56386962-56386983[+] | 0[0] | 0[0] | 3821[25] | 0[0] | 0[0] |
| KPNA2 | chr17:66042934-66042955[+] | 0[0] | 0[0] | 0[0] | 0[0] | 967[17] |
| BSG | chr19:582808-582829[+] | 0[0] | 0[0] | 520[13] | 0[0] | 0[0] |
| PPP1R11 | chr6:30037491-30037512[+] | 0[0] | 0[0] | 131[5] | 0[0] | 0[0] |
| LRRC59 | chr17:48458821-48458842[-] | 0[0] | 0[0] | 0[0] | 0[0] | 117[5] |
| WTAP | chr6:160176682-160176702[+] | 0[0] | 0[0] | 0[0] | 0[0] | 307[8] |
| HOXB3 | chr17:46626680-46626687[-] | 0[0] | 0[0] | 0[0] | 4[3] | 4[7] |
| SEC23IP | chr10:121693243-121693263[+] | 0[0] | 0[0] | 0[0] | 0[0] | 36[5] |
| MLEC | chr12:121135534-121135555[+] | 0[0] | 0[0] | 420[5] | 0[0] | 0[0] |
| MLEC | chr12:121137273-121137294[+] | 0[0] | 0[0] | 522[5] | 0[0] | 0[0] |
| ZNF664 | chr12:124499878-124499899[+] | 0[0] | 0[0] | 0[0] | 0[0] | 106[6] |
| ZCCHC3 | chr20:279570-279591[+] | 0[0] | 0[0] | 64[5] | 0[0] | 0[0] |
| TM9SF2 | chr13:100215013-100215023[+] | 0[0] | 0[0] | 0[0] | 0[0] | 310[6] |
| C22orf30 | chr22:32080893-32080915[-] | 0[0] | 0[0] | 0[0] | 0[0] | 42[5] |
| CDC23 | chr5:137523620-137523641[-] | 0[0] | 0[0] | 128[6] | 0[0] | 0[0] |
| PCNP | chr3:101312996-101313017[+] | 0[0] | 0[0] | 117[7] | 0[0] | 0[0] |
| INSIG1 | chr7:155100112-155100133[+] | 0[0] | 0[0] | 1006[8] | 0[0] | 0[0] |
| ARID5B | chr10:63856255-63856262[+] | 0[0] | 0[0] | 0[0] | 367[5] | 373[6] |
| LOC647979 | chr20:34637300-34637322[-] | 0[0] | 0[0] | 0[0] | 0[0] | 105[6] |
| ZNF238 | chr1:244219804-244219825[+] | 0[0] | 0[0] | 626[17] | 0[0] | 0[0] |
| PTGFRN | chr1:117531842-117531863[+] | 0[0] | 0[0] | 54[5] | 0[0] | 0[0] |
| AMMECR1L | chr2:128622581-128622602[-] | 0[0] | 0[0] | 210[5] | 0[0] | 0[0] |
| PAWR | chr12:79986098-79986119[-] | 0[0] | 0[0] | 0[0] | 0[0] | 71[5] |
| UBAP2L | chr1:154235830-154235851[+] | 0[0] | 0[0] | 296[6] | 0[0] | 0[0] |
| RAP1BL | chr12:69053673-69053693[+] | 0[0] | 0[0] | 0[0] | 0[0] | 306[9] |
| KIAA1522 | chr1:33238868-33238875[+] | 433[15] | 0[0] | 0[0] | 433[15] | 433[15] |
| IRF2BP2 | chr1:234741945-234741951[-] | 0[0] | 0[0] | 0[0] | 42[7] | 42[7] |
| ORAI1 | chr12:122079742-122079763[+] | 0[0] | 0[0] | 232[5] | 0[0] | 0[0] |
| C1QDC1 | chr12:30862533-30862540[-] | 0[0] | 0[0] | 0[0] | 229[7] | 0[0] |
| PUS7L | chr12:44122975-44122996[-] | 0[0] | 0[0] | 63[6] | 0[0] | 63[6] |
| CBLL1 | chr7:107401383-107401403[+] | 0[0] | 0[0] | 0[0] | 0[0] | 182[6] |
| MCL1 | chr1:150549633-150549654[-] | 0[0] | 0[0] | 603[6] | 0[0] | 0[0] |
| REST | chr4:57799684-57799691[+] | 421[5] | 0[0] | 0[0] | 0[0] | 0[0] |
| PEF1 | chr1:32095505-32095512[-] | 0[0] | 0[0] | 0[0] | 102[5] | 122[8] |
| KLHL15 | chrX:24002172-24002193[-] | 0[0] | 0[0] | 421[7] | 0[0] | 0[0] |
| TGFBR1 | chr9:101915920-101915939[+] | 0[0] | 0[0] | 0[0] | 0[0] | 141[5] |
| RAB21 | chr12:72180727-72180748[+] | 0[0] | 0[0] | 69[5] | 0[0] | 0[0] |
| RREB1 | chr6:7251587-7251607[+] | 0[0] | 0[0] | 0[0] | 0[0] | 2922[5] |
| HN1 | chr17:73132212-73132232[-] | 0[0] | 0[0] | 0[0] | 0[0] | 7363[6] |
| LDLR | chr19:11242802-11242823[+] | 0[0] | 0[0] | 724[10] | 0[0] | 0[0] |
| EPHA7 | chr6:93951073-93951093[-] | 0[0] | 0[0] | 0[0] | 0[0] | 145[10] |
| KLF13 | chr15:31666782-31666803[+] | 0[0] | 0[0] | 235[9] | 0[0] | 0[0] |
| LIX1L | chr1:145498975-145498994[+] | 0[0] | 0[0] | 0[0] | 0[0] | 121[7] |
| HMGA2 | chr12:66359589-66359611[+] | 0[0] | 0[0] | 0[0] | 0[0] | 2031[18] |

**Table S6** The association of IGF1R expression with clinicopathological

characteristics of PCa patients

| Variables | Cases  (n) | IGF1R | | *P* value |
| --- | --- | --- | --- | --- |
|  |  | High | Low |  |
| Total | 291 | 154 | 137 |  |
| *Age (years)* |  |  |  |  |
| ≥60 | 175 | 85 | 90 |  |
| <60 | 116 | 69 | 47 | 0.073 |
| *Gleason score* |  |  |  |  |
| 5-7 | 163 | 94 | 69 |  |
| 8-10 | 128 | 60 | 68 | 0.076 |
| *Pathologic T stage* |  |  |  |  |
| T1/T2 | 246 | 137 | 109 |  |
| T3/T4 | 45 | 17 | 28 | 0.034 |
| *Pathologic N stage* |  |  |  |  |
| Negative | 240 | 132 | 108 |  |
| Positive | 51 | 22 | 29 | 0.164 |
| *Pathologic M stage* |  |  |  |  |
| Negative | 290 | 154 | 136 |  |
| Positive | 1 | 0 | 1 | 0.471 |

**Table S7** Cox regression analysis of IGF1R expression as survival predictor

| Variables | Univariate Cox regression analysis | |  | Multivariate Cox regression analysis | |
| --- | --- | --- | --- | --- | --- |
|  | RR (95% CI) | *P* value |  | RR (95% CI) | *P* value |
| *Age (years)* |  |  |  |  |  |
| <60 vs. ≥60 | 1.276 (0.212 to 7.690) | 0.791 |  | NA | NA |
| *Gleason score* |  |  |  |  |  |
| 5-7 vs. 8-10 | 8.999 (0.957 to 84.629) | 0.055 |  | NA | NA |
| *Pathologic T stage* |  |  |  |  |  |
| T3+T4 vs. T1+T2 | 3.105 (0.281 to 34.260) | 0.355 |  | 4.279 (0.384 to 47.683) | 0.237 |
| *Pathologic N staging* |  |  |  |  |  |
| Positive vs. Negative | 9.321 (0.845 to 102.813) | 0.068 |  | NA | NA |
| *Pathologic M stage* |  |  |  |  |  |
| Positive vs. Negative | NA | NA |  | NA | NA |
| *IGF1R expression* |  |  |  |  |  |
| High VS. Low | 7.290 (0.737 to 72.137) | 0.089 |  | 7.947 (0.816 to 77.405) | 0.074 |

NA: not analyzed
